# Retraction Note: Effects of hPMSCs on granulosa cell apoptosis and AMH expression and their role in the restoration of ovary function in premature ovarian failure mice

**DOI:** 10.1186/s13287-022-03183-6

**Published:** 2022-10-13

**Authors:** Hongqin Zhang, Qianqian Luo, Xueyan Lu, Na Yin, Dongli Zhou, Lianshuang Zhang, Wei Zhao, Dong Wang, Pengchao Du, Yun Hou, Yan Zhang, Wendan Yuan

**Affiliations:** 1 School of Basic Medical Sciences & Institute of Reproductive Diseases, Binzhou Medical University, Yantai, 264003 Shandong China; 2 School of Basic Medical Sciences, Binzhou Medical University, Yantai, 264003 China; 3 Health School of Laiyang, Laiyang, 265200 China; 4 State Key Laboratory of Stem Cell and Reproductive Biology, Institute of Zoology, Chinese Academy of Sciences, Beijing, 100101 China

## Retraction Note: Stem Cell Research & Therapy (2018) 9:20 10.1186/s13287-017-0745-5

The Editors-in-Chief have retracted this article at the authors' request. After publication, concerns were raised regarding partial overlap between Figs. 2 and 5–7 in this article and Figs. 1 and 3–5, respectively, in an article by some of the same authors that was submitted and published within a close time frame [1]. The authors have confirmed that some incorrect images had been used in Figs. 5 and 6. The authors have repeated the H&E and immunohistochemistry experiments to address these concerns, but were unable to retrieve the original western blot data. The Editors-in-Chief therefore no longer have confidence in the data presented here.

All authors agree to this retraction.

## References

[1] Yin N, Wang Y, Lu X (2018). hPMSC transplantation restoring ovarian function in premature ovarian failure mice is associated with change of Th17/Tc17 and Th17/Treg cell ratios through the PI3K/Akt signal pathway. Stem Cell Res Ther.

